# Usefulness of Noninvasive Predictors of Oesophageal Varices in Black African Cirrhotic Patients in Côte d'Ivoire (West Africa)

**DOI:** 10.1155/2012/216390

**Published:** 2012-07-22

**Authors:** Alassan Kouamé Mahassadi, Fulgence Yao Bathaix, Constant Assi, Aboubacar Demba Bangoura, Emile Allah-Kouadio, Henriette Ya Kissi, Abdoulaye Touré, Stanislas Doffou, Issa Konaté, Alain Koffi Attia, Mathieu Benoit Camara, Thérèse Aya Ndri-Yoman

**Affiliations:** ^1^Service d'Hépato-Gastroentérologie, CHU de Yopougon, 21 BP 632 Abidjan 21, Cote d'Ivoire; ^2^Service d'Hépato-Gastroentérologie, CHU de Cocody, Abidjan, Cote d'Ivoire; ^3^Service d'Imagérie Médicale, CHU de Yopougon, Abidjan, Cote d'Ivoire; ^4^Service d'Imagérie Médicale, CHU de Cocody, Abidjan, Cote d'Ivoire

## Abstract

*Aims.* To determine the usefulness of platelet count (PC), spleen diameter (SD) and platelet count/spleen diameter ratio (PC/SD ratio) for the prediction of oesophageal varices (OV) and large OV in black African patients with cirrhosis in Côte d'Ivoire. *Materials and Methods.* Study was conducted in a training sample (111 patients) and in a validation sample (91 patients). *Results.* Factors predicting OV were sex: (OR = 0.08, *P* = 0.0003), PC (OR = 12.4, *P* = 0.0003), SD (OR = 1.04, *P* = 0.002) in the training sample. The AUROCs (±SE) of the model (cutoff ≥ 0.6), PC (cutoff < 110500), SD (cutoff > 140) and PC/SD ratio (cutoff ≤ 868) were, respectively; 0.879 ± 0.04, 0.768 ± 0.06, 0.679 ± 0.06, 0.793 ± 0.06. For the prediction of large OV, the model's AUROC (0.850 ± 0.05) was superior to that of PC (0.688 ± 0.06), SD (0.732 ± 0.05) and PC/SD ratio (0.752 ± 0.06). In the validation sample, with PC, PC/SD ratio and the model, upper digestive endoscopy could be obviated respectively in 45.1, 45.1, and 44% of cirrhotic patients. Prophylactic treatment with beta blockers could be started undoubtedly respectively in 36.3, 41.8 and 28.6% of them as having large OV. *Conclusion.* Non-invasive means could be used to monitor cirrhotic patients and consider treatment in African regions lacking endoscopic facilities.

## 1. Introduction

Oesophageal varices (OV) due to portal hypertension are a major concern in cirrhotic patients because of the risk of bleeding and related high mortality [1]. The prevalence of OV in newly diagnosed cirrhotic patients is approximately 60–80% and the 1-year rate of first variceal bleeding is approximately 5% for small OV and 15% for large OV [1, 2]. The determination of the presence of OV by upper digestive endoscopy is therefore mandatory in patients with cirrhosis at diagnosis [3].

For long-term followup, guidelines recommend monitoring of cirrhotic patients by routine endoscopy for the detection of the development of OV and to initiate prophylactic measures to prevent the bleeding of OV when they become large [3, 4]. Endoscopy is however a costly, invasive, and time-consuming procedure [5]. 

It is obvious that in most African countries monitoring cirrhotic patients with endoscopy even at baseline or during followup is a challenge for clinicians due to the lack or not widely implemented and accessible endoscopy units [6]. 

Several studies have reported that platelet count (PC), spleen diameter (SD) and their ratio (PC/SD), portal vein diameter, and Child-Pugh score were strongly associated with the presence of OV in cirrhotic patients [7–10]. Predictive models derived from these parameters were therefore constructed as surrogate tools for the prediction of OV or large OV [10–19]. None of these noninvasive means have been validated in black African patients with cirrhosis. 

In sub-Saharan countries of Africa as in Côte d'Ivoire, the burden of cirrhosis and hepatocellular carcinoma is high, mainly due to viral hepatitis B and C [20, 21]. The detection of OV in African patients with cirrhosis is a matter of concern because of the frequent malfunction or unavailability of endoscopy units [6]. Validation of these surrogate means is therefore needed.

Recently non-invasive markers of liver fibrosis have been studied for the prediction of OV and large OV mainly in patients with cirrhosis related to viral hepatitis C and alcohol consumption. Some of them need complicated calculation or are not routinely available in most peripheral hospitals in Côte d'Ivoire [22].

The main objective of this study was to determine factors associated with OV and the diagnostic accuracy of PC, SD, and PC/SD ratio to predict OV and large OV in cirrhotic patient in Côte d'Ivoire.

## 2. Material and Methods

### 2.1. Patients

Two hundred two Ivorian black patients (111 patients of the training sample and 91 patients of the validation sample) were recruited consecutively in two teaching hospitals of Abidjan (Yopougon and Cocody), the economic capital of Côte d'Ivoire from January to December 2009. All of them had cirrhosis without any previous variceal bleeding, OV band ligation, or sclerotherapy. None of them had had primary prophylactic treatment for variceal bleeding or any surgical treatment for portal hypertension at inclusion. Patients with hepatic schistosomiasis, chronic malaria, liver abscess, abdominal tuberculosis, hepatocellular carcinoma hematologic malignancies, and sickle cell anaemia were excluded [23–27]. Cirrhosis was diagnosed by the means of physical, laboratory, and abdominal ultrasonography and computerized tomography examinations [28, 29]. Laparoscopy was performed on patients with any clinical, biologic, or radiologic signs suggestive of cirrhosis, and diagnosis of cirrhosis was retained when the surface of the liver showed nodularity and irregularity suggestive of cirrhosis [30]. All patients underwent at admission, clinical, radiologic and laboratory examinations. Viral aetiology of cirrhosis was considered when one of these serological tests of viral hepatitis B (hepatitis B surface antigen, hepatitis B core antibody) or C (hepatitis C antibody) was positive. Alcoholic aetiology was made when patient's declared alcohol consumption was more than 50 g per day when measurable or local alcohol beverage consumption was three times per day in the past five years and correlated with biological abnormalities related to alcohol consumption [31]. Laboratory examination comprised liver function tests (transaminases, alkaline phosphatase, gamma-glutamyl transferase, total bilirubin, prothrombin time, and albumin) that allowed classifying cirrhotic patients according to the Child-Pugh score: class A: score 5-6, class B: score 7–9, and class C: score: 10–15 [28].

### 2.2. First Part of the Study

The training sample included 111 (median age: 49 years, range: 62 years) cirrhotic patients from the Teaching Hospital of Yopougon. Among these patients, 29.7% were female. All of them underwent endoscopy at diagnosis performed by the senior staff members of the gastroenterology unit with high skills on endoscopy procedures using optic and video endoscopes (GIF XQ 10 Olympus and EG-250W5 Fujinon) to determine the presence and the stage of OV. OV were graded according to the French classification system derived from the Japan Research Society for Portal Hypertension system which comprises 3 stages as follows [32]:

 Stage 1: small OV that flatten with insufflation and not confluent.
 Stage 2: tortuous OV not confluent and occupying less than one-third of the oesophagus lumen.
 Stage 3: tortuous OV confluent occupying more than one-third of oesophagus lumen.

Large OV was considered when stage of OV was greater than stage 1.

Ultrasonographic assessment of spleen and portal vein diameters were carried out in supine position with suspended inspiration. Imaging for each patient was preformed by one senior radiologist using a 3.5 Mhz transducer (Toshiba Nemio 30, Tokyo Japan). The SD was measured when the coronal image was obtained viewing the spleen on its long axis from the diaphragm to its inferior side. The portal vein was scanned by placing the transducer in transversal oblique position depicting its long axis. Its diameter was measured at its widest point just distal to the union of the splenic and superior mesenteric vein as recommended by others [33, 34]. 

### 2.3. Second Part of the Study

Predictive values obtained in the training sample were then validated in 91 cirrhotic patients (35.8% females, median age: 50 years) from the Teaching Hospital of Cocody. All of them met the inclusion criteria as mentioned above and underwent a clinical and laboratory examination. Endoscopic assessment of OV was performed with optic and video endoscopes (Olympus GIF XQ 20, GIF V70) by the staff member of the gastroenterology unit using the same 3 stages classifications of OV and adopted by the Ivorian Society of Gastroenterology and Digestive Endoscopy (SIGEED). Patients were classified according the Child-Pugh score. SD and portal vein diameter were assessed by the same procedure described above and by senior radiologists of the Teaching Hospital of Cocody using HITACHI EUD 525 sonograph (Tokyo, Japan).

### 2.4. Statistical Analysis

The endpoints were the prediction of OV and large OV in the training sample. Categorical or ordinal variables were expressed as number and percentage and continuous variables as median and range. Chi square or Fischer's test, if appropriate, was used to compare categorical variables and Mann-Withney's test for continuous variables. Correlation of SD, PC, and PC/SD ratio with the stage of OV was done using Spearman's rho test. Post-hoc analysis (with significant level set at 0.01) of differences of these parameters between stages of OV was performed by analysis of variance with Dunnett's T3 test for group comparison [35]. Predictive models of the presence of OV and large OV were constructed using backward logistic regression analysis. The diagnostic values of the model, SD, PC, and PC/SD ratio were determined by calculating the area under the receiver operating characteristic curve (AUROC) and the best cut-point that maximized the sensitivity and specificity was selected. The diagnostic accuracy was expressed as sensitivity, specificity, positive and negative predictive values, and positive likelihood ratio. Multiple comparisons of AUROCs were done using the Mac Henley method and procedures described by Hanley and McNeil [36], J. N. Mandekar and S. Mandekar [37]. In the validation sample, cutoff of PC, SD, and PC/SD ratio and predictive models obtained from the training sample were applied and numbers of correctly classified cirrhotic patients with OV or large OV were reported. All statistical analysis were two tailed and carried out with SPSS v16 (Chicago, IL) and SAS v9. 1 (SAS Institute Inc., Cary, NC, USA).

## 3. Results

### 3.1. Training Sample

#### 3.1.1. Patient's Characteristics

**Figure 1:**
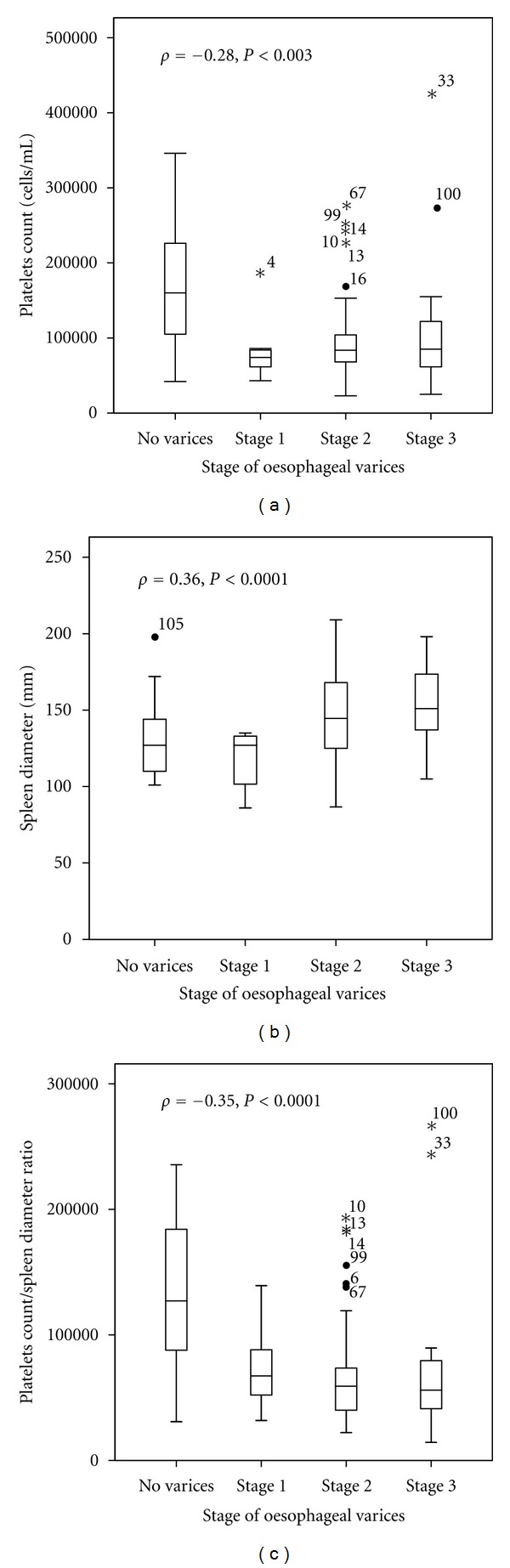
Box plots of platelets count (a), spleen size (b), and platelets count to spleen size ratio (c) according to the stage of oesophageal varices. The box represents the interquartile range; the top and the bottom of the box are, respectively, the 25th and 75th percentile. The line across the box is the median. The lower and upper values are indicated by the whiskers. Stars and circles represent the outliers and extreme values.

Characteristics of cirrhotic patients of the training sample are summarized in Table 1. Among these patients, 84 (75.7%) had decompensated cirrhosis with ascites and 47 (42.3%) were in Child-Pugh class C. The prevalence of OV was 76.6% (95% CI: 67.4–83.9). Among those with OV, 78 (92%) had LOV. Cirrhosis was related to chronic hepatitis B, C, and alcohol consumption in 61.3%, 12.6%, and 20.7%, respectively. Mixed aetiologies were marginal. The stage of OV correlated positively with the SD (rho = 0.36, *P* < 0.0001) and negatively with PC (rho = −0.28, *P* = 0.003) and PC/SD ratio (rho = −0.35, *P* < 0.0001). As illustrated in Figure 1, cirrhotic patients with no OV or stage 1 OV tended to have high level of PC and PC/SD and low SD than those with large OV (≥stage 2). Post-hoc comparisons showed that PC/SD ratio was greater in cirrhotic patients with no OV than those with stage 2 (*P* < 0.0001) and stage 3 (*P* = 0.001) but quite similar to that of patients with stage 1 OV (*P *= 0.03, significance level sets at 0.01). In univariate analysis variables significantly associated with the presence of OV were sex (*P *= 0.002), PC (*P *< 0.0001), SD (*P *= 0.002), and PC/SD ratio (*P *< 0.0001). For further analysis, PC were dichotomised by its median value (<93 000 and ≥93 000).

#### 3.1.2. Prediction of OV

**Figure 2:**
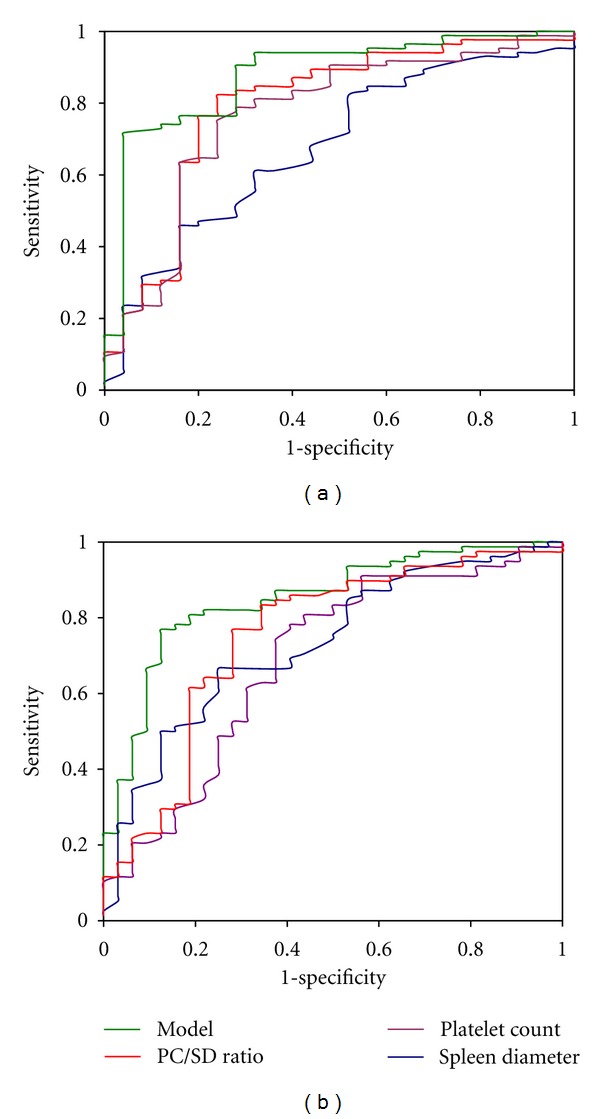
Receiver operating characteristic curve for the prediction of oesophageal varices (a) and large oesophageal varices (b) of the model, platelet count, spleen diameter, and platelet count spleen diameter ratio in the training sample.

Factors predicting the presence of OV in multivariate analysis (Table 2) were sex (OR = 0.08, *P* = 0.0003), PC (OR = 12.4, *P* = 0.0003), and SD (OR = 1.04, *P* = 0.002). The regression function was −2.5 × sex (female = 0, male = 1) + 2.5 × platelet count ((cells/mm^3^), 1 = platelet count < 93000 and 0 = platelet count ≥ 93000) + 0.04 × Spleen diameter (mm) − 4.2. The AUROCs (±SE) of the model, PC, SD, and PC/SD ratio were, respectively, 0.879 ± 0.04, 0.768 ± 0.06, 0.679 ± 0.06, and 0.793 ± 0.06 (Table 3). The accuracy of model in predicting the presence of OV was superior to that of PC (*P* = 0.03) and SD (*P *= 0.001) but similar to that of PC/SD ratio (*P *= 0.06) after pairwise comparisons of their respective AUROCs. PC, SD, and PC/SD showed similar accuracy in predicting the presence of OV (Figure 2). However, high rates of diagnostic accuracies (77.5, 81.1 and 87.3%, resp.) were obtained with PC (cutoff < 110500 cells/mm^3^), PC/SD (cutoff ≤ 868), and the model (cutoff ≥ 0.6). The concordance kappa between the model and PC or PC/SD ratio was 0.5 (*P* < 0.0001) and 0.6 (*P *< 0.001). Among the 111 cirrhotic patients in the training sample, 22.5, 18.9, and 12.7% were, respectively, misclassified by PC, PC/SD ratio, and the model. The distribution of these patients according to the stage of OV was, respectively, no OV: 8(7.2%), 6(5.4%), 8(7.3%); OV stage 1: 1(0.9%), 2(1.8%), 0(0%); stage 2: 9(8.1%), 10(9.0%), 4(3.6%); stage 3; 7(6.3), 3(2.7%), 2(1.8%). 

#### 3.1.3. Effect of the Prognosis of Cirrhosis on the Prediction of OV

Diagnostic accuracies for the prediction of OV were similar between the model, PC, and PC/SD whatever the prognosis of cirrhosis (results not shown). (Cochran-Mantel-Haenszel chi-square *Q* = 0.19, df = 2, *P* = 0.9).

#### 3.1.4. Prediction of Large OV

**Figure 3:**
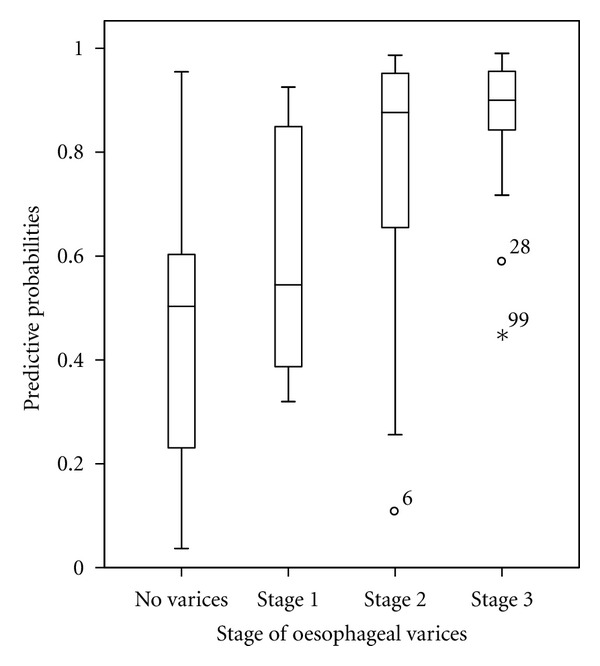
Box plots of predicted probabilities of large oesophageal varices by the model including 4 parameters: sex, spleen diameter, platelet count, and Child-Pugh score. The box represents the interquartile range; the top and the bottom of the box are, respectively, the 25th and 75th percentile. The line across the box is the median. The lower and upper values are indicated by the whiskers. Stars and circles represent the outliers' and extremes' values.

Factors predicting large OV in multivariate analysis (Table 2) were sex (OR = 0.11, *P *= 0.001), PC (OR = 0.25, *P* < 0.0001), SD (OR = 1.05, *P *= 0.04), and child-Pugh class B (0R: 0.2, *P *= 0.04). The regression function was −2.1 × sex (female = 0, male = 1) + 1.4 × platelet count ((cells/mm^3^), 1 = platelet count < 93000 and 0 = platelet count ≥ 93000) + 0.04 × spleen diameter (mm) −1.6 × Child-Pugh class B −0.7 × Child-Pugh class C (reference: Child-Pugh class A) −4.2. In this setting, the accuracy of the model including 4 parameters was superior to that of PC (*P* = 0.005), SD (*P *= 0.02), and PC/SD ratio (*P *= 0.05). However, the accuracy of PC/SD ratio was superior to that of PC (*P *= 0.004) but similar to that of SD (*P *= 0.8) (Figure 2). With the model, cirrhotic patients with large OV (stage 2 or 3) were more likely to have high predictive probabilities than those with no OV or stage 1 OV (Figure 3). Hence, a predictive probability cutoff of ≥0.6 yielded a sensitivity, specificity, and positive predictive value of 82.1, 62.5, and 84.2%, respectively, to ascertain the presence of large OV in cirrhotic patient with an AUROC of 0.850. Using PC, SD, and PC/SD ratio, the prediction of large OV was good with a positive predictive value greater than 80% (Table 3). However, 23.6, 27, 33.4, and 21.6% of cirrhotic patients in the training sample were, respectively, misclassified by the model, PC, SD, and PC/SD ratio. The number of cirrhotic patients with large OV missed by these noninvasive means was 14(12.7%), 17(15.3%), 29(26.1%) and 12(10.8%), respectively.

#### 3.1.5. Application in the Validation Sample

Characteristics of cirrhotic patients in the validation sample are resumed in Table 1. Among them, 66(72.5%) and 67(73.6%) had, respectively, clinical ascites and cirrhosis related to viral hepatitis B or C. The prevalence of OV was 79.1% and 60 (65.9%) patients had large OV. Cirrhotic patients of the training and validation sample were similar regarding age, sex, total bilirubin, PC, and the prevalence of OV. However, cirrhotic patients of the validation sample were less graded in Child-Pugh class C (*P *= 0.01), had high values of prothrombin time (*P *= 0.02), low values of albumin (*P *< 0.001), SD (*P *< 0.001), and portal vein diameter (*P *< 0.001) compared to that of training sample. 

## 4. Diagnosis of OV Using the Model, PC, and**** PC/SD Ratio

**Figure 4:**
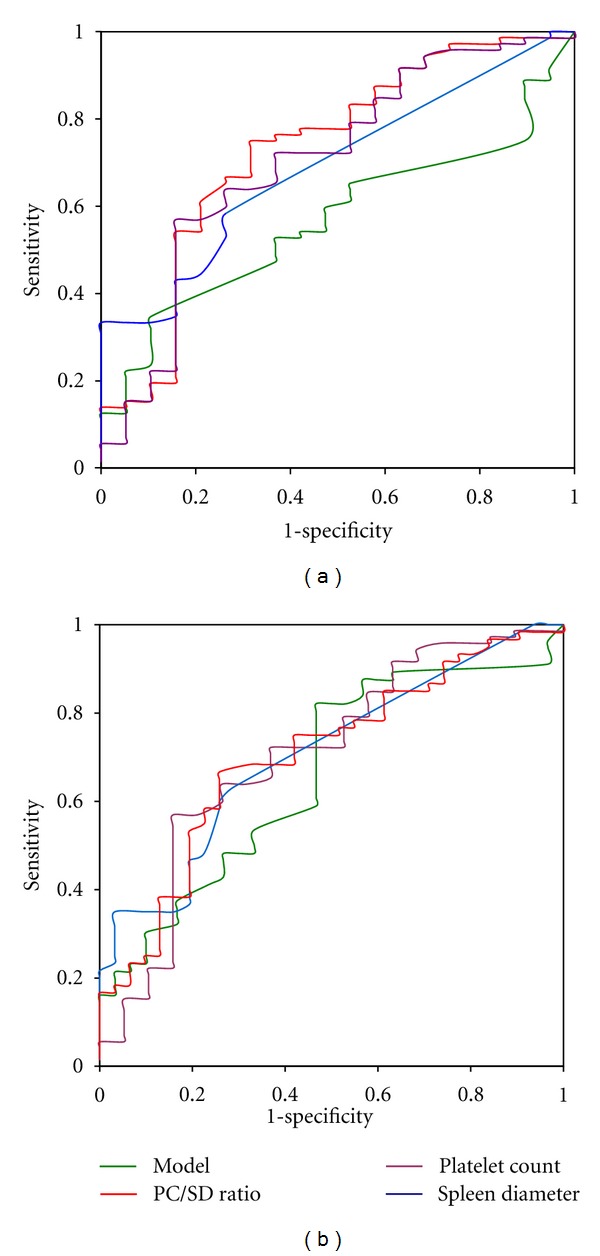
Receiver operating characteristic curve for the prediction of oesophageal varices (a) and large oesophageal varices (b) of the model, platelet count, spleen diameter, and platelet count spleen diameter ratio fitted in the validation sample.

Fitting the model in the validation sample yielded an AUROC statistically significant but lower than that of training sample (0.568 ± 0.07 versus 0.879 ± 0.06 *P *< 0.0001). Pairwise comparisons of AUROCs did not show any difference between the model (0.568 ± 0.07), PC (0.705 ± 0.07), SD (0.691 ± 0.06), and PC/SD ratio (0.730 ± 0.07) for the prediction of OV in the validation sample (Figure 4). However, SD was not suitable to distinguish cirrhotic patients with OV or large OV in the validation sample. The cutoff obtained in the training sample was lower than the median value of SD of cirrhotic patients in the validation sample.

Applying their respective cutoff, the specificity, positive, and predictive value and diagnostic accuracies in the validation sample were 52.6%, 82%, and 56% for the model; 84.2%, 93.2%, and 62.6% for PC, 79%, 90.9%; 60.4% for PC/SD ratio. The number of false positive and false negative patients were 9 (9.9%) and 31(34.1%) for the model, 3(3.3%) and 31(34.1%) for PC, and 4(4.4%) and 32(35.2%) for PC/SD ratio. Endoscopy could be obviated, respectively, in 45.1%(41/91), 45.1%(41/91), and 44%(40/91) with PC; PC/SD ratio, and the model.

## 5. Diagnosis of Large OV Using the Model with Four Parameters, PC, and PC/SD Ratio

The AUROC of the model fitted in validation sample was statistically significant but lower than that of the training sample (0.662 ± 0.06 versus 0.850 ± 0.04; *P *= 0.001). However, in the validation sample, pairwise comparisons of AUROCs (Figure 4) did not show any difference between the model (0.662 ± 0.06), PC (0.666 ± 0.06), SD (0.708 ± 0.05), and PC/SD ratio (0.730 ± 0.06) for the prediction of large OV (Figure 4). A cutoff ≥ 0.6 the model yielded a diagnostic accuracy of 53.8% with a specificity of 74.2% and positive predictive value of 76.5%. Among 34(37.4%) predicted as having large OV by the model, 26(28.6%) were confirmed and 8(8.8%) were false positive (no OV: *n *= 5, stage 1 OV *n *= 3). PC (cutoff < 106500 cells/mm^3^) yielded a diagnostic accuracy of 63.7%, with a specificity of 80.7%, and positive predictive value of 84.6%. Among 36 (39.6%) of 91 predicted as having large OV by PC, 33(36.3%) were correctly classified and 6(6.6%) were false positive (no OV: *n* = 3, stage 1 OV: *n* = 3). With PC/SD ratio (cutoff ≤ 897), the performance was quite similar to that of PC reaching a diagnostic accuracy of 67%, a specificity of 74.2%, and positive predictive value of 82.6%. Among 46(50.5%) predicted as having large OV, 38(41.8%) were correctly classified and 8(8.8%) were false positive (no OV: *n* = 4, stage 1 OV: *n* = 4). Overall, using PC, PC/SD ratio, and the model, the proportions of cirrhotic patients in the validation sample in which prophylactic treatment could be started undoubtedly while awaiting upper digestive endoscopy were, respectively, 36.3, 41.8 and 28.6%.

## 6. Discussion

Our study suggests that in Côte d'Ivoire, factors associated with the presence of OV in cirrhotic patients were sex, PC, and SD. The model and PC/SD ratio were equivalent in terms of prediction of OV in Ivorian patients with cirrhosis regarding their respective AUROCs in the training sample. However, in the validation sample, the diagnostic accuracies of the model, PC, and PC/SD ratio were similar regardless of the prognosis of cirrhosis. This suggests that in countries with low level of medical infrastructures such as Côte d'Ivoire, these noninvasive means could be used to predict the presence of OV in cirrhotic patient. Furthermore, the presence of large OV could be predicted by a model combining factors predicting the presence of OV and factors assessing liver function determined by Child-Pugh score. The model yielded a sensitivity of 82.1% and positive predictive value of 84.2%. However, in the validation sample, the accuracy of the model for the prediction of OV or large OV was lower than that of PC and PC/SD ratio. This discrepancy was probably related to confounding effect of Child-Pugh score. In fact, cirrhotic patients in the training sample had more advanced liver disease than those in the validation sample.

Several studies have focused on determining clinical, laboratory, and radiologic factors associated with the presence of OV or large OV and their diagnostic accuracies in clinical practice when used as a single parameter or combined in a model. Giannini et al. [11] found that PC/SD ratio yielded high diagnostic accuracy to predict the presence of OV. This was confirmed by Agha et al. [17], using the same cutoff of 909. Zaman et al. [10] found that PC and Child-Pugh score were factors associated with the presence of OV, whereas low PC and palpable spleen or radiologic splenomegaly were accurate to predict the presence of large OV as demonstrated by others [14, 15]. These studies however included patients with various aetiologies of cirrhosis mainly related to hepatitis C virus or alcohol ingestion. Hong et al. [38], demonstrated in Asian patients with cirrhosis only due to hepatitis B virus infection that spleen width and portal vein diameter were factors independently associated with the presence of OV. Our finding suggests that factors underlying the onset of OV in Ivorian cirrhotic patients were probably modulated by gender. Regarding OV or large OV, women were less at risk compared to men. The difference between the model predicting OV (including sex, PC, and SD) and PC/SD ratio, however, did not reach statistical significance. This could be probably related to the small size of our training sample.

PC or PC/SD ratio, respectively, used as a single parameter was a good predictor of OV or large OV in our study and negatively correlated with the stage of OV. In contrasting to the finding of Sebastiani et al. [22], PC maintained high positive predictive value either for the prediction of OV or large OV in our study. However, the overlapping values of PC according to the stage of OV depicted in Figure 1, explained the various cutoffs of PC and PC/SD ratio reported previously and in our study [10, 16, 22, 39]. Our result, which is not consistent with that of Hong et al. [38], suggests that thrombocytopenia remains a reliable predictor of OV as explained by the well-known mechanism involving hypersplenism and low production of thrombopoietin by hepatocytes due to fibrosis in the liver [40, 41]. 

We found in our study that patients with mild liver failure (those in Child-Pugh class B) were at less risk of having large OV, and those with advanced liver failure (those in Child-Pugh class C) had no risk of having large OV, compared to those with quiescent cirrhosis (those in Child-Pugh class A), adjusted to other factors. This finding emphasized the peculiarity of the natural history of cirrhosis in Ivorian cirrhotic patients regarding the influence of Child-Pugh score on the risk of having large OV as reported by others [11, 42]. One plausible explanation is the probable role of toxic effect of herbal remedies frequently prescribed to cirrhotic patients by traditional healers in Africa that might result in liver decompensation even in those with quiescent cirrhosis [43]. These traditional medications acted as confounders in estimating the influence of prognostic factors on the risk of presence of large OV. In fact, most of our cirrhotic patients were admitted in hospital with decompensated cirrhosis due to the use of herbal medicines.

Furthermore, the lower diagnostic accuracy of SD in our study suggests that splenomegaly in the African context might not be useful as predictor of OV. In fact chronic parasitism and anaemia, more prevalent in Africa due to various aetiologies, might result in splenomegaly misleading the diagnostic accuracy of the SD [44]. To some extent, this may explain the finding that the portal vein diameter in our study was not significant to predict OV as previously demonstrated by others [9, 38]. This is probably related to an underlying portal hypertension which could increase the size of portal vein.

This study has been carried out in black African patients with cirrhosis mostly related to hepatitis B and C, living in Côte d'Ivoire where the facilities to perform upper digestive endoscopy routinely is very limited. Therefore, our finding suggests that other means could be used to monitor patients with cirrhosis at diagnosis in some areas of Côte d'Ivoire where access to medical facilities is limited. PC, PC/SD, and the model were accurate to predict the presence of OV and showed high sensitivity and positive predictive value of up to 80%. Each of these noninvasive means may be used routinely according to the availability of appropriate medical equipment for their determination. In fact, for the prediction of the presence of OV, the use of PC, PC/SD, or the model could obviate upper digestive endoscopy in almost 45% of cirrhotic patients in our study. However, the degree of accuracy of PC may be hampered by alcohol consumption as known factor of thrombocytopenia [45]. Therefore, PC/SD ratio which is more predictable must be used first in those with alcoholic cirrhosis as demonstrated by Giannini et al. [11].

The prevention of variceal bleeding is an important goal to be achieved in cirrhotic patients with large OV by implementing prophylactic treatment [3]. In our study, this treatment could be started in almost 30% of cirrhotic patients when these noninvasive means (model, PC, and PC/SD ratio) of prediction of large OV were applied although these patients have to undergo upper digestive endoscopy for confirmation.

This might be a step forward in the care of cirrhotic patients in developing countries particularly in Côte d'Ivoire where the malfunction of endoscopy units is frequently encountered.

The results of this study could not be extrapolated to include all cirrhotic patients in Côte d'Ivoire. In fact, those who had already bled or undergone prophylactic treatment for OV bleeding or those with hepatic schistosomiasis that constitute an important population of the patients attending our hepatology unit were not included. The diagnosis of cirrhosis in our study was mainly based on clinical, biologic, radiologic, and laparoscopic examinations. This method of diagnosis without any histologic examination of the liver may be less accurate as other causes of portal hypertension leading to OV could be missed. The variability of the ultrasonographic examination of the liver and spleen was another source of error regarding the results of the measurement of the spleen and portal vein diameters between the training and validation samples in our study. However, this study was strengthened by the characteristic differences between the validation and training samples both drawn from two distinct hospitals in Abidjan. It suggests that our finding could be reasonably applicable to other newly diagnosed cirrhotic patients in Côte d'Ivoire wherever their location in the country may be. However, upper digestive endoscopy remains the most reliable means for the detection of OV.

In conclusion, despite the lower diagnostic accuracy of the models in the validation sample, the prediction of OV or large OV by PC, PC/SD, ratio and models was good to be used as part of tools to monitor cirrhotic patients and consider treatment in geographical areas lacking endoscopic facilities. However, upper digestive endoscopy remains the more reliable means to monitor cirrhotic patients. Other studies should be initiated to validate the diagnostic accuracy of these noninvasive parameters in other African countries. 

## Figures and Tables

**Table 1:** Characteristics of cirrhotic patients of the training and validation samples.

	Training sample				Validation sample (*n* = 91)
	All (*n* = 111)	NOV (*n* = 26)	OV (*n* = 85)	*P* value
Age (years) [median (range)]	49 (62)	52 (52)	49.5 (63)	0.3	50 (59)
Sex (female) [*n* (%)]	33 (29.7)	14 (56)	19 (22.4)	0.002	35 (38.5)
Collateral venous circulation [*n* (%)]	24 (21.6)	4 (15.4)	20 (23.5)	0.3	5 (5.5)
Clinical ascites [*n* (%)]	84 (75.7)	21 (80.8)	63 (74.1)	0.3	66 (72.5)
Total bilirubin (mg/dL) [median (range)]	17.8 (247)	13 (248)	14 (234)	0.9	7 (247)
Prothrombin time (%) [median (range)]	55 (86)	67 (82)	56 (86)	0.2	64 (79)
Albumin (g/L) [median (range)]	26 (37)	25 (29)	25 (34)	0.3	22 (25)
Platelet count (×10^3^/mL) [median (range)]	930 (404)	146 (491)	90 (553)	0.0001	113 (553)
Spleen diameter (mm) [median (range)]	139 (123)	110 (123)	136 (124)	0.002	101 (123)
Portal vein diameter (mm) [median (range)]	12.8 (14)	11.8 (7)	12 (14)	0.8	10 (8)
PC/SD ratio [median (range)]	651.5 (2523)	1390 (4921.2)	642.8 (5530)	0.0001	880 (5530)
Child-Pugh score [*n* (%)]				0.4	
A	25 (22.5)	4 (15.4)	21 (24.7)		18 (19.8)
B	39 (35.1)	12 (46.2)	27 (31.8)		48 (52.7)
C	47 (42.3)	10 (38.5)	37 (43.5)		20 (22.0)
Oesophageal varices [*n* (%)]					
No varices	26 (23.4)	—	—		19 (20.9)
Stage 1	7 (6.3)	—	—		12 (13.2)
Stage 2	54 (48.6)	—	—		34 (37.4)
Stage 3	24 (21.6)	—	—		26 (28.6)
Aetiologies of cirrhosis [*n* (%)]^a^				0.3	
HBV	68 (61.3)	16 (61.5)	52 (61.2)		53 (58.2)
HCV	14 (12.6)	4 (15.4)	10 (11.8)		14 (15.4)
HBV + HCV coinfection	2 (1.8)	0	2 (20)		—
Alcohol	23 (20.7)	6 (23.1)	17 (20)		24 (26.4)
Mixed (viral and alcohol)	4 (3.6)	0	4 (4.7)		—

NOV: no oesophageal varices, OV: oesophageal varices, PC/SD ratio: platelet count-spleen diameter ratio, HBV: hepatitis B virus, HCV: hepatitis C virus

^a^Aetiologies were grouped in 2 classes: viral and others (viral and/or alcohol).

**Table 2:** Logistic regression of factors predicting the presence of oesophageal varices or large oesophageal varices.

	beta (SE)	Adjusted OR (95% CI)	*P*
Factors predicting OV			
Gender	−2.5 (0.7)		0.003
Male		1	
Female		0.1 (0.02–0.3)	
Platelet count (cell/mm^3^)	2.5 (0.7)		0.0003
≥93000		1	
<93000		12.4 (3.2–47.7)	
Spleen size (mm)	0.04 (0.01)	1.04 (1.01–1.07)	0.002
Factors predicting large OV			
Gender	−2.1 (0.6)		0.001
Male		1	
Female		0.1 (0.03–0.4)	
Platelet count (cell/mm^3^)	1.4 (0.5)		0.01
≥93000		1	
<93000		4.0 (1.4–11.72)	
Spleen size (mm)	0.04 (0.01)	1.05 (1.02–1.1)	<0.0001
Child-Pugh score			<0.0001
Class A		1	
Class B	−1.6 (0.8)	0.2 (0.04–0.9)	
Class C	−0.7 (0.8)	0.5 (0.1–2.3)	

Intercept of model predicting OV = −4.2, C-index: 0.878, *R* ^2^ = 0.31, *P* < 0.0001.

Intercept of model predicting LOV = −4.2, C-index: 0.850, *R* ^2^ = 0.30, *P* < 0.0001.

Beta: coefficient estimates, OR: odds ratio, SE: standard error, CI: confidence interval.

Platelet count dichotomized by the median value because of large value of coefficient estimates of platelet count (−10^-5^) when used as continuous variable not corrected by the exact method, Logistic regression analysis was computed on 110 patients due to one missing value in those with no OV.

**Table 3:** Diagnostic performances of non invasive means for the prediction oesophageal varices and large oesophageal varices in the training sample.

	AUROC ±SE	Cut-off	TP (*n*)	TN (*n*)	SS (%)	SP (%)	PPV (%)	NVP (%)	DA (%)	LR+
Oesophageal varices										
Platelet count	0.768 ± 0.06	<110500	68	18	80	69	89.5	51	77.5	2.6
Spleen diameter	0.679 ± 0.06	>140	44	18	51.8	72	86.3	30.5	55.9	1.9
PC/SD ratio	0.793 ± 0.06	≤868	70	20	82.4	76.9	92.1	57.1	81.1	3.6
Regression function	0.879 ± 0.04	0.3	82	7	96.5	28	82	70	80.9	1.3
		0.4	81	7	95.3	28	81.8	63.6	80	1.3
		0.5	80	10	94.1	40	84.2	66.7	81.8	1.6
		0.6	79	17	92.9	68	90.8	73.9	87.3	2.9
		0.7	69	18	81.2	72	90.8	52.9	79.1	2.9
		0.8	62	18	72.9	72	89.9	43.9	72.7	2.6
Large oesophageal varices										
Platelet count	0.688 ± 0.06	<106500	61	20	78.2	60.6	82.4	54.1	73	2
Spleen diameter	0.732 ± 0.05	>137	49	25	62.8	46.3	86	46	66.7	1.2
PC/SD ratio	0.752 ± 0.06	≤897	66	21	84.6	63.6	84.6	61.8	78.4	2.3
	0.3	75	9	96.2	28.1	76.5	75	76.4	1.3
Regression function	0.850 ± 0.04	0.4	73	11	93.6	34.4	77.7	68.8	76.4	1.4
		0.5	68	15	87.2	46.9	80	60	75.5	1.6
		0.6	64	20	82.1	62.5	84.2	58.8	76.4	2.2
		0.7	59	25	75.6	78.1	89.4	56.8	76.4	3.5
		0.8	51	27	65.4	84.4	91.1	50	71	4.2

AUROC: area under receiver operating characteristic curve, SE: standard error, PC/SD ratio: platelet count-spleen diameter ratio, TP: true positive TN: true negative SS: sensitivity SP: specificity PPV: positive predictive value NVP: negative predictive value DA: diagnostic accuracy, LR+: positive likelihood ratio, Prevalence of OV: 76.6%, logistic regression analysis was computed on 110 patients due to one missing value in those with no OV.
